# *Xenopus* Oocyte’s Conductance for Bioactive Compounds Screening and Characterization

**DOI:** 10.3390/ijms20092083

**Published:** 2019-04-27

**Authors:** Amani Cheikh, Hager Tabka, Yassine Tlili, Andrea Santulli, Noureddine Bouzouaya, Balkiss Bouhaouala-Zahar, Rym Benkhalifa

**Affiliations:** 1 Laboratoire Venins et Molécules Thérapeutiques, Institut Pasteur de Tunis, Université Tunis El Manar, 13 Place Pasteur BP74, Tunis 1002, Tunisia; tabkahager@yahoo.fr (H.T.); yessinetlili24@gmail.com (Y.T.); balkiss.bouhaouala@pasteur.tn (B.B.-Z.); 2 Laboratorio di Biochimica Marina ed ecotossicologia, Dipartimento di Scienze della Terra e del Mare, Università degli Studi di Palermo, 91100 Trapani, Italy; andrea.santulli@unipa.it; 3 BiotechPole Sidi Thabet, Ariana 2020, Tunisie; noureddine@bouzouaya.com; 4 Faculté de Médecine de Tunis, Université Tunis El Manar, 15 Rue Djebel Lakhdhar, La Rabta, Tunis 1007, Tunisia

**Keywords:** *Xenopus* oocyte INa^+^, astaxanthin, marine natural products, bioactive compounds

## Abstract

Background: Astaxanthin (ATX) is a lipophilic compound found in many marine organisms. Studies have shown that ATX has many strong biological properties, including antioxidant, antiviral, anticancer, cardiovascular, anti-inflammatory, neuro-protective and anti-diabetic activities. However, no research has elucidated the effect of ATX on ionic channels. ATX can be extracted from shrimp by-products. Our work aims to characterize ATX cell targets to lend value to marine by-products. Methods: We used the *Xenopus* oocytes cell model to characterize the pharmacological target of ATX among endogenous Xenopus oocytes’ ionic channels and to analyze the effects of all carotenoid-extract samples prepared from shrimp by-products using a supercritical fluid extraction (SFE) method. Results: ATX inhibits amiloride-sensitive sodium conductance, xINa, in a dose-dependent manner with an IC50 of 0.14 µg, a maximum inhibition of 75% and a Hill coefficient of 0.68. It does not affect the potential of half activation, but significantly changes the kinetics, according to the slope factor values. The marine extract prepared from shrimp waste at 10 µg inhibits xINa in the same way as ATX 0.1 µg does. When ATX was added to the entire extract at 10 µg, inhibition reached that induced with ATX 1 µg. Conclusions: ATX and the shrimp Extract inhibit amiloride-sensitive sodium channels in *Xenopus* oocytes and the TEVC method makes it possible to measure the ATX inhibitory effect in bioactive SFE-Extract samples.

## 1. Introduction

ATX is a ketocarotenoid in a variety of living organisms, many of which are found in the marine environment [1]. Marine sources of ATX include 6–8 million tons of shrimp waste produced annually [2] representing an important natural source [3]. SFE-Extract is a crude marine extract obtained from shrimp by-products by supercritical CO2. We are interested in discovering the relationship between the presence of ATX and its pharmacological effect.

The current literature describes the potential beneficial effects of ATX on cancer, diabetes, cardiovascular disease, gastrointestinal diseases, liver diseases, neurodegenerative diseases, and skin diseases thanks to its antioxidant properties [1,4,5,6]. However, there are few studies of metabolic pathways and molecular targets involved in ATX antioxidant properties [1]. What’s more, multiple studies have reported the involvement of calcium [7,8], potassium [9,10,11,12], sodium [13], and chloride channels [14] in the development of pathologies in which oxidative stress plays a major role. These channels represent very important pharmacological targets for several pathologies. More research on the involvement of ion channels in oxidative stress-associated human diseases may help find both new early-detection tools and novel therapeutic strategies.

Today, the *Xenopus* oocyte is widely recognized and used for the expression and characterization of exogenous receptors, ionic channels and transporters [15], and homologies have been reported between *Xenopus* oocytes ionic channels and neuronal and muscle mammal subtypes [16]. In fact, *Xenopus* oocytes display their own ionic channels on the cell surface and different studies have demonstrated the presence of voltage-gated potassium, sodium and calcium channels in addition to chloride conductance, calcium-activated potassium conductance, other receptors and trans-membrane proteins [17]. These are all involved in different physiological activities during oogenesis and different cell cycle stages. In fact, these oocytes are commonly used to explore the effects of the components of venom on endogenous or exogenous expressed ionic channels [18,19,20]. Recently, we demonstrated the improvements of oocytes in studying and characterizing the effects of venom fractions on particular endogenous conductance [21].

Assuming that ATX can affect ion channels, our objective was twofold: first, we sought to determine the subtype of ion channels that ATX recognizes, and second, we wished to use the *Xenopus* oocyte ionic channels cell model to carry out pharmacological studies.

To achieve this twin goal, we analyzed the pharmacological effect of ATX and SFE-Extract separately under different experimental conditions and then simultaneously for a better understanding and elucidation of their pharmacological targets.

## 2. Results

Xenopus oocytes extracted from the South African clawed frog *Xenopus laevis,* the most commonly used model for studying the properties, organization, and cellular roles of ionic channels and transporters [22]. The oocyte is the cellular precursor for the development of specialized cells. To characterize the presence of ATX in crude extracts, we explore the effect of pure ATX and/or SFE-Extract on endogenous currents, both separately and concomitantly. We recall that SFE-Extract is a crude marine extract obtained from shrimp by-product by supercritical CO2.

### 2.1. Pharmacological Characterization of ATX Activity on Xenopus Oocytes Endogenous Currents

First, we improve experimental conditions to better record the profiles of *Xenopus* oocytes endogenous currents. Two media compositions and record conditions were tested: (I) OR_2_ medium which has been previously used for sodium channel studies [23] (ii) BAMS solution which is recommended for voltage gated calcium channels isolation and makes it possible to record sodium currents recording.

**Figure 1:**
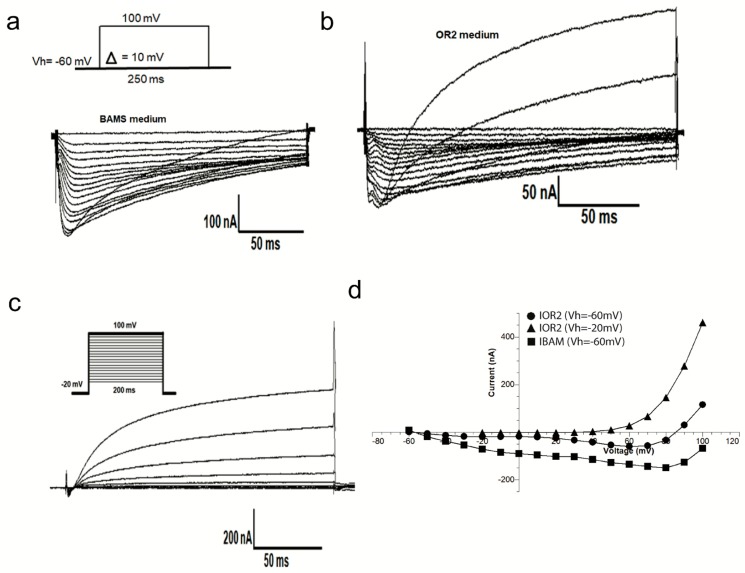
*Xenopus* oocytes current recordings obtained in different conditions of bathing media and holding potentials. The same oocyte was used for the experiments in each panel. Representative current traces of *Xenopus* oocytes held at −60 mV in BAMS medium (Na^+^ = 50 mM) (**a**), in OR_2_ medium (Na^+^ = 82.5 mM) (**b**), and in OR_2_ medium at Vh = −20 mV (**c**). Current-voltage relationships constructed using the current sizes of a, b and c families at different potential steps (**d**).

Figure 1a,b shows how the medium and the holding potential values could affect recorded currents in the presence of OR_2_ or BAMS medium. We notice that currents recorded in the OR_2_ medium, at Vh = −60 mV, are twice as low as those recorded in a BAMS solution. In addition, in OR_2_ at Vh = −20 mV, only an outward current is recorded in *Xenopus* oocytes (Figure 1c). According to the I-V relationship (Figure 1d), in the OR_2_ medium and at Vh= −60 mV, xIend progressively activates (from membrane potential value of −30 mV) before reaching a maximum value of −80 nA at +60 mV and then inactivates slowly to reverse at a potential value of +90 mV. This emphasizes the recording conditions recommended by [23]. This data encouraged us to use the OR_2_ medium and −60 mV as a holding potential, for a more selective isolation of sodium conductance’s and to carry out better pharmacological studies.

The effect of ATX, on *Xenopus* oocytes endogenous currents was subsequently studied using an OR_2_ medium. Current-voltage (I–V) relationships were obtained from records generated in the range of −60 to + 100 mV stimulations.

Currents were induced from a holding potential of −60 mV with potential steps of 10 mV increments during 250 ms.

**Figure 2:**
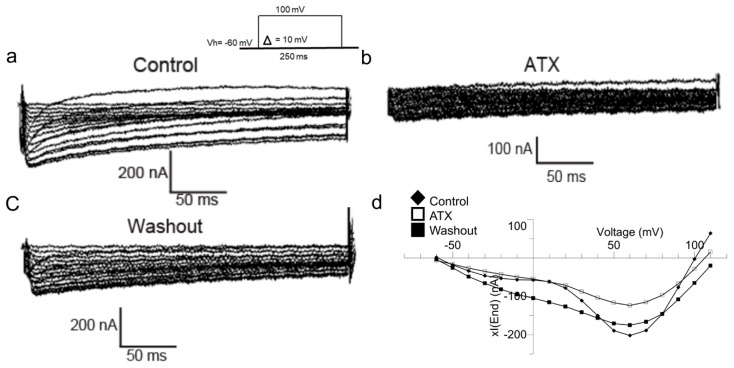
ATX inhibits Endogenous currents in *Xenopus* oocytes. TEVC currents recorded with [Na^+^] (82.5 mM) in the bath. Current families traces in control conditions (**a**), in the presence of ATX (**b**) and after washout (**c**). Current-voltage relationships constructed using the current sizes of a, b and c families at different potential steps (**d**).

Figure 2 shows that endogenous currents amplitudes are reduced in the presence of ATX (1 µg) and the washout allows a partial recovery of them. These results suggest that ATX inhibits *Xenopus* oocytes endogenous currents in a reversible manner. For the most thorough pharmacological characterization of the type of current that ATX has inhibited we carried out tests in the presence of tetrodotoxin (TTX) and amiloride. TTX and amiloride, are both sodium channels inhibitors, with greater specificity of TTX in fast inactivating voltage activated sodium channels, and amiloride in slow inactivating sodium channels.

**Figure 3:**
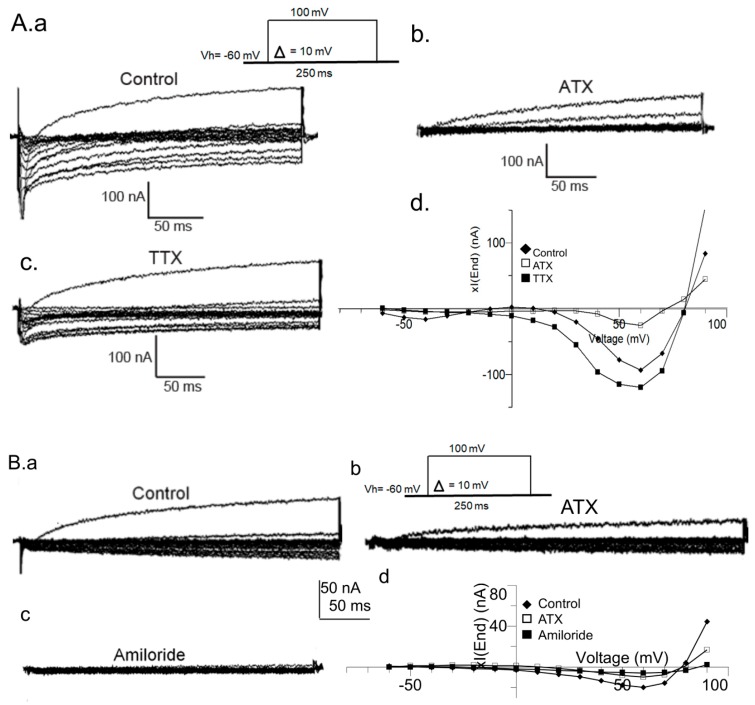
ATX inhibits voltage gated sodium channels that are sensitive to amiloride in *Xenopus* oocytes. TEVC currents were recorded with [Na^+^] (82.5 mM) in the bath. In (**A**) Current traces families in control conditions (**a**) in the presence of ATX (**b**) with ATX removed and replaced by TTX (**c**). Current-potentials relationships constructed from a, b and c show that ATX inhibits the current amplitude contrary to TTX (**d**). In (**B**) Current families trace in control conditions (**a**) in the presence of ATX (**b**) and then in presence of amiloride (**c**). Current-potentials relationships constructed from a, b and c show that ATX inhibits the current amplitude in the same manner as amiloride (**d**).

Figure 3A demonstrates that TTX (1 µM) in b does not affect the family of xIend (a), contrary to ATX (1 µg) which inhibits the current amplitude at all potential values (c) as elucidated in the I-V relationship (d). However, ATX inhibits xIend just as amiloride (10 µM) (Figure 3B(a–c)). This inhibition is reported by the I–V curves (d) suggesting that ATX has an inhibitory effect on amiloride-sensitive endogenous voltage gated sodium channels.

### 2.2. Pharmacological Characterization of SFE-Extract on Xenopus Oocytes’ Endogenous Currents

SFE-Extract was prepared from a *P. longirostris* by-product and used to purify ATX. To validate the pharmacological assay on *Xenopus* oocytes’ endogenous currents described above, we characterized ATX presence in a crude marine extract (SFE-Extract).

**Figure 4:**
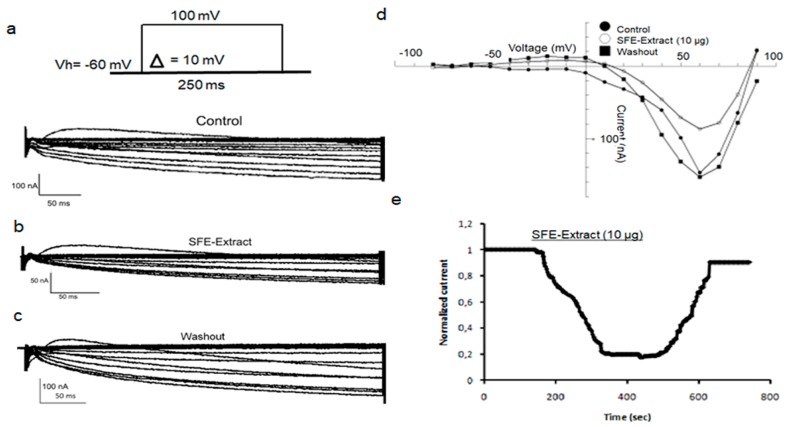
Reversible inhibitory effect of SFE-Extract on endogenous currents in *Xenopus* oocytes in OR2 and BAMS media. In OR2, the currents were recorded according to the protocol above in the control conditions (**a**) in the presence of SFE-Extract (10 µg) (**b**) after washout (**c**). I–V relationships are plotted in (**d**). Oocytes held at −80 mV in BAMS solution containing CsOH, 2 mM to block potassium currents. The oocytes were then depolarized from −40 to +100 mV during 300 ms. Current time course inhibition of SFE-Extract, 10 μg. Maximal effect occurs about 2 min after the beginning of the perfusion. A partial recovery from the inhibitory effect was observed after the washout of the Extract (**e**).

Figure 4 shows the effect of the SFE-Extract on *Xenopus* oocytes’ endogenous currents obtained in OR2 medium (Figure 4a–d). Current-voltage (I–V) relationships were taken from records generated in the range of −80 to + 100 mV stimulations and were induced from a holding potential of −60 mV, with potential incremental steps of 10 mV during 300 ms. Figure 4a,b shows the inhibitory effect of SFE-Extract (10 µg) on *Xenopus* oocytes’ endogenous currents (xIend). The washout allows the amplitude current recovery (traces in c) as illustrated by the IV-curves relationships in Figure 4d. To emphasize the SFE-Extract inhibitory effect, other series of experimentations were carried out in a BAMS medium as presented in the plot (e) SFE-Extract inhibits 79.91% ± 5.93% of xIend and the washout allows the current to recover at 90.36% ± 5.62% (*n* = 4, *p* < 0.05).

In conclusion, the SFE-Extract inhibitory and reversible effect on endogenous currents is validated in both media—OR2 and BAMS.

### 2.3. Comparison of ATX Versus SFE-Extract Effects on Endogenous Amiloride-Sensitive Sodium Current

As a function of ATX and/or SFE-Extract concentrations, we study the relationship between compound(s) concentration(s) and endogenous currents amplitudes recorded in an OR2 medium and as xINa endogenous currents. Table 1 and Table 2 show that ATX 0.1 µg inhibits 34.83 ± 0.06% of xINa while 0.25 µg inhibits 40.25 ± 0.10% and 55.75 ± 0.14% inhibition is obtained at 0.5 µg. Maximum inhibition of 65.99 ± 0.17% is reached at 1 µg. On the other hand, SFE-Extract (10 µg) inhibits only 36.46 ± 0.05% of amiloride-sensitive xINa. Interestingly, this inhibitory effect reaches 66.51 ± 0.07% when SFE-Extract (10 µg) is enriched by ATX (1 µg).

**Figure 5:**
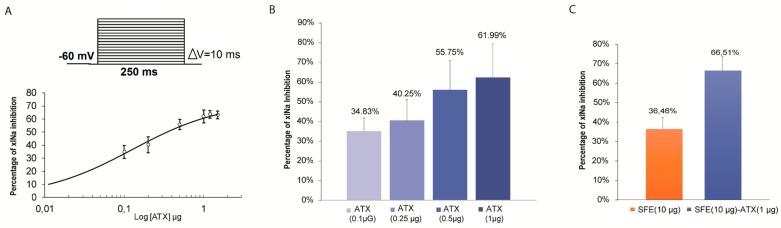
Analysis of ATX and SFE-Extract inhibitory effects on amiloride-sensitive sodium currents in *Xenopus* oocytes. In (**A**) Doses-response curve for the M1 inhibitory effect on Xenopus endogenous current traces recorded during a 250 ms voltage clamp step at 60 mV from a HP of −60 mV. (**B**) Histograms illustrating the ATX inhibitory effect of the current peak at different concentrations. In (**C**) Histograms of the percentage of inhibition in the presence of SFE-Extract (10 µg) alone and enriched by ATX 1 µg.

A dose-response curve in Figure 5A is plotted within the range 0.1 µg to 1 µg of ATX with a simple Hill function. The averaged curve obtained from 3 to 7 oocytes shows the maximal of inhibition of xINa at 75.70 ± 13.63% with an IC50 of 0.14 µg ± 0.078 and a Hill coefficient of 0.68 ± 0.27. 

Histograms in Figure 5B,C compare the variation of inhibition percentages of xINa amplitudes at different ATX concentrations in the presence of SFE-Extract or in both SFE-Extract plus ATX. This interesting result shows that the inhibition induced by SFE-Extract would result at least in part by the presence of ATX.

### 2.4. Electrophysiological Characterization of ATX Versus SFE-Extract on Endogenous Amiloride-Sensitive Sodium Channels

**Figure 6:**
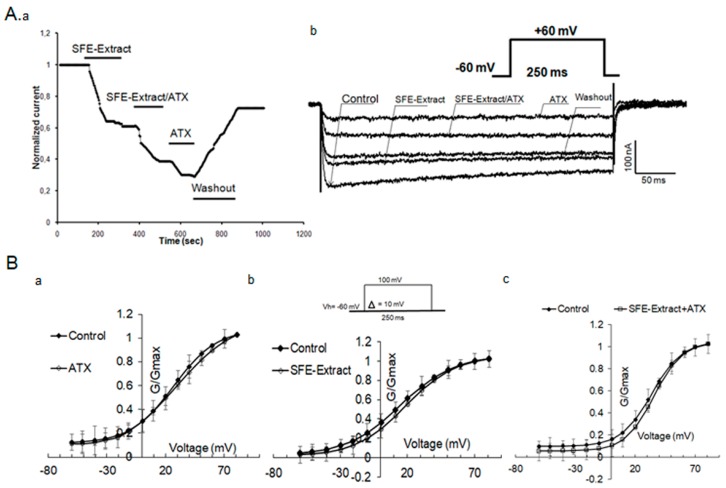
Characterization of ATX and SFE-Extract effects on sodium currents activation in *Xenopus* oocytes. In **(A**) Time course inhibition in the presence of SFE-Extract alone, the extract enriched with ATX, and then the ATX alone. Maximal effect is obtained in the presence of ATX and the washout allows a partial recovery of the current (**a**). Representative traces of xINa recorded at +60 mV in control conditions and in the presence of SFE-Extract and SFE-Extract enriched with ATX (1 µg) (**b**). In (**B**) Mean steady state activation curves of xINa in control conditions, in the presence of ATX (**a**) SFE-Extract (10 µg) (**b**) and SFE-Extract/ATX (**c**). Data are means ± SEM (*n* = 4 to 6) and fitted with a standard Boltzmann function.

Further experiments were conducted in the presence either of SFE-Extract enriched with ATX or pure ATX. Figure 6Aa shows the recorded currents. The current decreases gradually after 25 s of SFE-Extract perfusion and reaches a steady state within 4 min (36%). SFE-Extract replacement by SFE-Extract/ATX affects the current amplitude (65%) more, but the ATX-induced inhibition is the most important, reaching 83%.

After 4 min of washout with OR_2_, the current recovers nearly 75%, indicating that the effect of SFE-Extract is partially reversible. SFE-Extract enriched with ATX inhibits the xINa amplitude more than SFE-Extract (10 µg) alone (Figure 6Ab). The subsequent application of ATX (1 µg) alone acts in the same way as SFE-Extract enriched with ATX. The washout makes the major part of the recovery of the current amplitude possible.

Figure 6B shows the activation curves (g/g_max_) of amiloride-sensitive xINa in control conditions in the presence of ATX (1 µg) (a) in the presence of SFE-Extract (10 µg) (b) and of (SFE-Extract (10 µg) + ATX (1 µg)) (c). The Boltzmann function fitted curves show that the potential for half maximal activation (V_1/2_) in the presence of ATX, does n0t show a significant change compared to the control, the V1/2 is modified from 26.88 ± 3.72 to 29.14 ± 5.19 mV (*p* < 0.05). However, a significant change of the slope factor is observed changing from 18.52 ± 3.67 to 25.59 ± 5.55 under ATX. A comparable effect is obtained in the presence of SFE-Extract that changes the V1/2 in a non-significant manner from 15.99 ± 1.79 mV in control conditions to 18.52 ± 1.44 mV under SFE-Extract. When both ATX and SFE-Extract are simultaneously applied, the potentials of half activation and the voltage dependence slope factors show no significant change since in control V1/2 is 33.72 ± 1.79 mV and is 35.51 ± 1.24 mV under SFE-extract enriched with ATX. The slope factors are approximately the same: 12.68 ± 1.60 in control versus 11.89 ± 1.10 in the presence of both compounds.

The main purpose of the present investigation was to explore the pharmacological target of ATX among membrane ion channels. ATX could be purified from different natural sources, mainly the *Heamatococcus pluvialis* algae but also, from crustaceans such as *Parapenaeus longirostris*, i.e., shrimp wastes. ATX and shrimp extract are known for their powerful antioxidant activities. Our study demonstrates that ATX inhibits amiloride-sensitive sodium channels. In fact, ATX (1 µg) inhibits the recorded currents in the same manner as amiloride (10 µM), whether or not TTX is inactive.

## 3. Discussion

The principal purpose of the present investigation is to explore the pharmacological target of ATX among membrane ion channels. Using *Xenopus* oocyte endogenous currents, our results made it possible for us to evaluate the presence of similar ATX effects in shrimp extracts prepared by Supercritical Fluid Extraction. The powerful antioxidant activities of ATX and shrimp extracts are well known. Our study demonstrates that ATX inhibits amiloride-sensitive sodium channels. A clear qualitative correlation between SFE-Extract and ATX activities was demonstrated in extracts prepared from *P. longirostris* by-products by Supercritical Fluid Extraction at a concentration of 10 µg highlighting the presence of the ATX molecule at low concentrations.

To isolate sodium conductance in the *Xenopus* oocyte model, we used different media such as OR_2_. In fact, ATX affects sodium conductance in OR_2_ at a holding potential equal to −60 mV. It is noteworthy that at this value, inward activating and inactivating currents were recorded at potential ranges from −60 to +100 mV with a maximal amplitude of +50 or +60 mV. Current profiles reveal the atypical subpopulation profiles of slow voltage-gated sodium channels, sNa [23,24,25,26]. Previous publications indicate that this kind of subpopulation is induced by long-lasting depolarization and that only the membrane depolarization can activate sNa^+^ channels, suggesting that our recordings correspond to another subtype of VGSC. One class of Na^+^ channels is insensitive to tetrodotoxin (TTX) [23,26], while another Na^+^ current that is induced by long-lasting depolarization is sensitive to TTX. Other researchers have reported the existence of another class of Na^+^ channels that are active at resting potential independent of a depolarization that amiloride- and phenamil-sensitive (present in the oocytes of one in three animals) [16]. Therefore, in our experiments, amiloride-sensitive inward currents are present in an average of 15% of the tested oocytes.

Remarkably, ATX inhibition is reversible, dose-dependent and xINa amiloride-sensitive; it acts at a low concentration (0.1 µg, equivalent to 8 nM) with a maximum inhibition of 75.70 ± 13.63%, an IC50 of 0.14 ± 0.078 µg and a Hill coefficient of 0.68 ± 0.27. The voltage-dependency of activation (G_max_50) is not significantly affected: it is 26.88 ± 3.72 under control conditions and 29.14 ± 5.19 mV in the presence of 1 µg ATX. However, ATX has a more pronounced effect on curve steepness, proving the modification of channel conformation. For resveratrol and quercetin, the steady state activation is not changed [27]. ATX inhibits *Xenopus* oocytes amiloride-sensitive VGSC and affects their activation kinetics.

By using the TEVC method, we also, demonstrate an ATX-like activity in SFE-Extract consisting of an inhibitory effect on amiloride-sensitive voltage-gated sodium channels. We reported that SFE-Extract enriched with ATX is more active than SFE-Extract alone. The inhibition shifted from 36% to 66%, suggesting the probable presence of ATX at an active concentration and confirming the inhibitory role of the added ATX. However, the SFE-Extract/ATX effect on current amplitude decrease may also be attributed, at least in part, to enrichment with polyphenols that could also be active on the same conductance.

We highlighted the presence of the ATX molecule at low concentrations in extracts prepared from *P. longirostris* by-products by Supercritical Fluid Extraction at a concentration of 10 µg. SFE-Extract 10 µg acts in a manner comparable to ATX (0.5 µg), making possible to conclude that there is a clear qualitative correlation between SFE-Extract and ATX activities. As SFE-Extract is heterogeneous, we suggested checking how it affects endogenous currents in a BAMS medium to limit the interferences between the different compounds and the native conductance.

These results suggest that ATX inhibits sodium current that is sensitive to amiloride in *Xenopus* oocytes. Likewise, pharmacological studies with polyphenol antioxidants such as quercetin, catechin and resveratrol show peak INa blockade with IC50s of 19.4, 76.8 and 77.3 µM, respectively [27].

Mammalian species express nine functional voltage-gated Nav channels. Three of these—the cardiac-specific isoform Nav1.5 and the neuronal isoforms Nav1.8 and Nav1.9—are relatively resistant to TTX. Homologies were reported between *Xenopus* oocytes ionic channels and neuronal and muscle mammal subtypes [16]. Recent research has shown that the cardio-protective role of polyphenols may result from an interaction with ionic channels [22,28,29,30,31,32]. Wallace et al. [24] argue that several grape polyphenols like resveratrol directly inhibit cardiac voltage gated sodium channels in a dose-dependent manner and that the red grape extract significantly inhibits INav1.5 at 15 µg/mL. In cardiac cells, reactive oxygen species (ROS) can down-regulate cardiac sodium channels. ROS can also reverse this effect [33], in part via the down-regulation of SCN5A transcription [34] through protein kinase C (PKC) activation [35]. In the lungs, alveolar epithelial cells express two amiloride-sensitive Na^+^ channels at their apical. The antioxidant application responsible for reactive species decreases, reversing the diminution of amiloride-sensitive currents induced by ROS [36] via a pathway involving ERK1/2 [37]. By contrast, in the kidneys, a central role of oxidative stress in the pathophysiology of sodium retention was demonstrated. In addition, using patch-clamp experiments, H2O2 was shown to stimulate ENaC activity [38].

The inhibitory effect obtained with ATX on amiloride-sensitive xINa could result from a direct or indirect interaction between ATX and the channel. Antioxidants most often rather enhance the amplitude of the current, suggesting that under our experimental conditions, ATX switches to a pro-oxidant form. In fact, some Carotenoids can switch from antioxidants to pro-oxidants either as a function of carotenoid concentration or because of the high partial pressure of oxygen or carotenoid capacity to interact with and localize within membranes. Compared to other carotenoids, Astaxanthin presents oxygen as a combination of hydroxyl and carbonyl groups [39]. For this reason, we do not eliminate the direct interaction hypothesis between ATX and the channel given the ATX hydroxyl groups. Further investigation with ATX is clearly required to define the range of its antioxidant capacity in *Xenopus* oocytes cells in addition to other experimentation with specific inhibitors of the main enzymes involved in the different antioxidant pathways.

In conclusion, the astaxanthin results on the sodium currents of *Xenopus* oocytes suggests the merit of exploring its effect on mammal sodium channels, which are considered to be key actors in cardio-protective and nociceptive mechanisms.

## 4. Materials and Methods

### 4.1. Extract Preparation from Shrimp By-Products

Industrial by-products of *Parapenaeus longirostris* were used as the astaxanthin source. Astaxanthin from dried shrimp waste powder was extracted by Supercritical Fluid Extraction (SFE, Helix System Basic Model, Applied Separation Inc, Allentown, PA, USA) applying the parameters reported in De la Fuente et al., 2006 [40]. The astaxanthin content in SFE-Extract was determined spectro-photometrically as per Simpson and Haard (1985) and by high-performance liquid chromatography with diode-array (HPLC-DAD, Agilent Technologies, Waldbronn, Germany) [41].

### 4.2. Isolation and Culture of Oocytes

Experiments on *Xenopus* oocytes were carried out according to the European Community Council Directive (86/609/EEC) for experimental animal care and procedures. The protocol for handling animals and oocytes extraction was approved by the Pasteur Institute of Tunis Biomedical Ethic Committee (http://www.pasteur.tn/index.php?option=com_content&view=article&id=497&Itemid=797, Approval on 19 May 2018; approval code: 2018/39/I/LR16IPT08/V0). Experiments were performed using stage-V or VI *Xenopus laevis* oocytes according to [42]. The females were purchased from Xenopus Express (Lyon, France). Mature female *Xenopus laevis* were anaesthetized by immersion in a 0.17% solution of tricaine (ethyl*M*-aminobenzoate). Parts of ovaries were then surgically removed from the abdominal cavity and bathed in a calcium-free Modified Barth’s Solution (MBS: NaCl 88 mM, KCl 1 mM, MgCl_2_ 0.41 mM, NaHCO_3_ 2.4 mM, MgSO_4_ 0.82 mM, HEPES 10 mM, pH 7.4). Oocytes were defolliculated enzymatically by incubation in collagenases A and B (Boehringer Roche, Meylan, France) 2 mg/mL, then stage V and stage VI oocytes were selected after mechanical defolliculation using forceps. Oocytes were then incubated in MBS sterile solution: calcium-free MBS was added with CaCl_2_ 2.4 mM, Ca (NO3)_2_ 0.33 mM, Gentamycin (Sigma, St. Quentin Fallavier, France) 50 mg/L, pH 7.4.

Electrophysiological recordings were realized in BAMS (BaOH, 40 mM; CsOH, 2 mM HEPES, 4 mM; NaOH, 50 mM; pH 7.4) for better isolation of voltage gated calcium channels by blocking outward potassium channels with barium and chlore channels by the hydroxyde. The OR_2_ medium (NaCl 82.5 mM; KCl 2.5 mM; MgCl_2_ 1 mM; CaCl_2_ 1 mM; Na_2_HPO_4_ 1 mM; HEPES 5 mM; pH 7.4) needed to be used for sodium channel studies [23] with higher sodium concentrations. The incubation (without antibiotics) medium was renewed daily. Chemicals were purchased from Sigma (St. Quentin Fallavier, France) and applied externally by adding them to the superfusate.

### 4.3. Oocyte Treatment

Oocytes were maintained in MBS culture medium and moved in OR_2_ or BAMS medium 15 min before recording. Specific inhibitors were specifically chosen to distinguish the kind of conductance affected by ATX and SFE-Extract. They were added directly and slowly during recording at adequate concentrations (TTX at 1 µM, amiloride at 10 µM and cadmium at 1 mM) and during at least 1 min before testing ATX and SFE-Extract. Pure ATX from *Haematococcus pluvialis* was applied in a dose dependent manner at 0.25, 0.5, 0.75 and 1 µg (equivalent to 5 µg/mL) and SFE-Extract was used at 10 µg (equivalent to 50 µg/mL).

### 4.4. Electrophysiological Measurements

Electrophysiological recordings were performed using a two-electrode voltage clamp and a GeneClamp 500 amplifier (Molecular Devices, Union City, CA, USA). Oocytes were immersed in BAMS or OR_2_ mediums and impaled with 2 intracellular glass electrodes filled with 3M CsCl or 3 M KCl. The resistance of the pulled electrodes (P-97 puller; Sutter Instruments, Novato, CA, USA) was 1–2 MΩ. The perfusion system was controlled by a Manifold Solution Changer (MSC-200; Bio-Logic; Grenoble, France). Data acquisition and analysis were performed using Clampex and Clampfit from Pclamp10 software (Molecular Devices, Union City, CA, USA). Leak and capacitive currents were subtracted during analysis. The study of the effects of the different samples (ATX, SFE-Extract, SFE-Extract + ATX) was carried out on *Xenopus* oocytes recorded endogenous currents using 250 ms/300 ms or 500 ms depolarizing pulses from −60 to +120 mV at holding potentials of −80 or −60 mV.

### 4.5. Electrophysiological Results Analysis

Data were recorded and analyzed by the PClamp10 software. The activation curve was estimated in terms of relative conductance g/g_max_ (g = I/(V − V_inv_) with V_inv_ as the inversion potential. Curves were fitted by the Boltzmann equation: g/g_max_ = 1/(1 + exp[(E0.5 − Em)/k] where Em is the membrane potential, E0.5 is the half-activation potential, and k is the slope factor.

### 4.6. Statistical Analysis

The analysis and fitting of mean data points were conducted using Clampfit10 software. Statistical analysis was performed using Excel and the numerical data were expressed as mean ± SEM. Differences were tested using an unpaired two tailed Student’s *t*-test, assuming that the population follows a Gaussian distribution. Differences were assumed to be significant when *p* < 0.05.

## 5. Conclusions

This work emphasizes that ATX and SFE-Extract specifically inhibit voltage-gated sodium channels in *Xenopus* oocytes, although SFE-Extract from *P. longirostris* also acts on voltage-gated calcium channels. ATX seems to be the main component responsible for amiloride-sensitive sodium conductance inhibition in *Xenopus* oocytes. The electrophysiological study of the activation kinetics of ATX *versus* SFE-Extract and SFE-Extract enriched with ATX demonstrates that ATX inhibits in a dose dependent manner and can inhibit xINa at low concentrations (80 nM). SFE-Extract (10 µg) inhibits and in a significant manner. We demonstrate that ATX is pharmacologically active on the *Xenopus* oocyte sodium channel and validates an ATX-like activity in SFE-Extract, as shown by the percentage of inhibition reached when ATX is added to the SFE-Extract. Antioxidants are known to increase the activity of amiloride-sensitive sodium channels. For this reason, it is suggested that ATX switches to a pro-oxidant form in our experimental conditions. Furthermore, the ATX inhibitory effect modifies the channel activation mechanism by slowing its kinetics and affecting the channel conformation, which does not invalidate the hypothesis of a direct interaction between ATX hydroxyl groups and the sodium channel.

Exploring the role of astaxanthin on mammal voltage-gated channels could be very important. The TEVC electrophysiological method will be used for further effective pharmacological investigations of ATX and SFE-Extract.

## Figures and Tables

**Table 1:** Amiloride-sensitive sodium current amplitude variation as a function of ATX concentration (4–6 experiments).

ATX (µg)	0.1	0.25	0.5	1
xINa inhibition %	34.83 ± 0.06	40.25 ± 0.10	55.75 ± 0.14	65.99 ± 0.17

**Table 2:** Amiloride-sensitive sodium current amplitude variation as a function of SFE-Extract and/or ATX concentrations (4–6 experiments).

Product	Concentration (µg)	xINa Inhibition%
SFE-Extract	10	36.46 ± 0.05%
SFE-Extract/ATX	10/1	66.51 ± 0.07%
